# Deer Browsing Increases Stem Slenderness and Crown Irregularity and Modifies the Effects of Light Gradients on Architecture of Forest Tree Saplings

**DOI:** 10.1002/ece3.70837

**Published:** 2025-01-20

**Authors:** Cecilia Cacciatori, Anna Gazda, Jan Bodziarczyk, Kacper Foremnik, Aklilu B. Madalcho, Zbigniew Maciejewski, Remigiusz Pielech, Andrzej Tomski, Antoni Zięba, Tomasz Zwijacz‐Kozica, Jerzy Szwagrzyk

**Affiliations:** ^1^ Department of Forest Biodiversity University of Agriculture in Krakow Krakow Poland; ^2^ Department of Natural Resource Management Wolaita Sodo University Wolaita Sodo Ethiopia; ^3^ Roztoczański National Park Zwierzyniec Poland; ^4^ Institute of Botany Jagiellonian University in Krakow Poland; ^5^ Institute of Mathematics, University of Silesia in Katowice Katowice Poland; ^6^ Tatra National Park Zakopane Poland

**Keywords:** browsing pressure, crown irregularity, crown slenderness, light gradient, sapling slenderness

## Abstract

Browsing by ungulates is commonly assumed to target the upper parts of sapling crowns, leading to reduced vertical growth or even growth cessation. However, the extent to which browsing induces shifts in resource allocation toward lateral growth remains unclear. This study explores the impact of browsing intensity (BI) and light availability on the architectural traits of six temperate tree species, focusing on height‐diameter ratio (H/D), crown slenderness (CL/CW), and crown irregularity (CI) across sapling height classes. Browsing pressure and architectural responses varied across height groups, reflecting diverse adaptive strategies. BI was weakly but negatively correlated with sapling height, indicating that even tall saplings (> 2 m) experience browsing, particularly in the lower crown. H/D consistently increased with BI across all height classes, with stronger effects in medium and tall saplings. Light influenced H/D differently between browsed and unbrowsed saplings: unbrowsed saplings showed reduced H/D only under high light conditions, while browsed saplings exhibited consistent reductions regardless of light levels. CL/CW was negatively but insignificantly affected by BI. Light increased CL/CW in unbrowsed saplings across all height classes but decreased it in browsed short and medium saplings, suggesting a ‘pruning’ effect of browsing that altered competition dynamics. Species‐specific analysis of *Fagus sylvatica* revealed an increase in CL/CW with BI, reflecting unique adaptive responses. CI increased significantly with BI across all height classes, with the strongest effects in medium and tall saplings. Light reduced CI in browsed short saplings but had inconsistent effects on unbrowsed individuals. Variation partitioning showed that light explained most variation in H/D and CL/CW for shorter saplings, while BI predominantly influenced CI in taller ones. By integrating the effects of browsing and light, this study provides insights into juvenile tree adaptations and resilience under ecological stressors, advancing our understanding of tree growth strategies in challenging environments.

## Graphical abstract

Ungulate browsing impacts both vertical and lateral growth of saplings, with effects varying by browsing intensity (BI), light availability, and sapling height class. BI increased crown irregularity (CI) and altered height‐diameter ratio (H/D), while light influenced crown slenderness (CL/CW) and mitigated CI in shorter saplings. This study highlights adaptive strategies of juvenile trees under ecological stressors, integrating the effects of browsing and light.

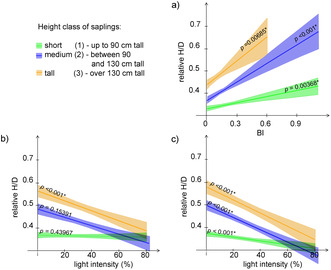

## 1 Introduction

Tree architecture has been defined as the visible expression of the genetic ground plant upon which the construction of a tree is based (Hallé and Oldeman 1970; Hallé, Oldeman, and Tomlinson 1978). However, despite being constrained by the underlying model inscribed in the species genome, tree architecture is constantly modified by exogenous factors (MacFarlane and Kane 2017). Because of that, it has a strong adaptive significance, being the result of a set of trade‐offs among different morpho‐physiological trait combinations to maximise species and individual fitness at given environmental conditions. In forest ecosystems, competitive interactions, herbivory, and randomly occurring disturbance events can select from the spectrum of allometric relationships allowed for a certain species the ones that best fit the changing set of local conditions (Walters et al. 2020), thus inducing a constant adjustment of tree architecture. The latter can be defined, and is treated in this study, as the result of the spatial arrangement of the structural components of a tree and the proportion in which these components stay among each other.

Environmental control on tree architecture is particularly important in the early ontogenetic tree stages. Seedlings and saplings of forest tree species grow in the highly challenging microenvironmental conditions of the understory, where access to light and nutrients is limited by the reduced light availability (Chazdon and Pearcy 1991) and the presence of competitors (Chen et al. 2010; Comita et al. 2010; Tyler and D'Antonio 1995). This is the crucial stage where species plasticity can decide their survival and fitness.

Out of the set of potential environmental drivers of juvenile tree architecture, light conditions have received the most attention so far. Indeed, light represents the key driver of morphological changes in woody species (Chazdon 1988; Claveau et al. 2002) and is the most heterogeneously distributed and dynamic factor in forest understory, due to the mosaic of canopy gaps of different sizes and shapes and to foliage distribution (Canham et al. 1994; Valladares and Guzmán 2006). In their study about the effect of light intensity on allometric traits of pine and spruce saplings in mountain forests, Ametzegui and Coll (2011) observed that light intensity negatively affected sapling slenderness and positively affected relative crown vertical length, while it did not show any effect on crown slenderness. Opposite results were reported by Orman et al. (2021), who, studying the impact of gap size and light intensity on juvenile individuals of *Abies alba* and *Fagus sylvatica* , found that light conditions did not affect trees slenderness, whereas increasing light availability negatively affected both crown length and crown area. Meanwhile, Bebre et al. (2020) found no response of height and structure of tree saplings to varying light conditions in a controlled shading experiment. While it is well‐ascertained that variations in light conditions induce major adjustments in tree architecture, other environmental factors, by directly constraining tree growth, can play an equally important role.

Browsing by ungulates is mainly regarded as a factor reducing growth rates of juvenile trees (Häsler, Senn, and Edwards 2007; Kupferschmid and Bugmann 2013; Kupferschmid, Wasem, and Bugmann 2014a, 2014b; Kupferschmid, Zimmermann, and Bugmann 2013; Zamora et al. 2001) and increasing their mortality (Ammer 1996; Canham et al. 1994; Gill 1992), thus exerting a huge impact on tree recruitment in forest ecosystems. In Europe, such impact has become stronger during the last decades, due to the considerable increase in red deer ( *Cervus elaphus* L.) population in most countries of the temperate region (Côté et al. 2004; Schulze et al. 2014). Shoots of woody plants are the major food item for deer, especially in winter (Churski et al. 2022). Browsing of the top shoots is especially detrimental, as it slows down the height growth of young trees and prevents them from escaping the herbivore pressure by growing beyond their reach (Renaud, Verheyden‐Tixier, and Dumont 2003). However, the effects of ungulate browsing on tree saplings may be evaluated not only in terms of juvenile tree height growth or individual mortality/survival. In fact, by the same process of selective biomass removal, browsing may also significantly affect sapling architecture and allometric proportions. Sapling height and slenderness, crown vertical and lateral development, length of branches, and leaf area are likely to respond to the impact of browsing. Yet, still little is known about the effects of the latter on juvenile tree architecture. In their review, Danell, Bergstrom, and Edenius (1994) synthesised the knowledge about the effects of browsing by moose on the architecture, biomass, and nutrients of woody plants, but they focused mostly on the last two aspects, and, when addressing architecture, they just discussed the effects of browsing on shoot length and branching. López‐Sánchez et al. (2021) analysed the response of young *Quercus ilex* individuals to cattle, sheep, and deer browsing in Spanish traditional silvopastoral systems. Churski et al. (2022) examined the tendency of tree saplings to create “cages” in response to high browsing pressure, thus focusing on three‐dimensional branching arrangement patterns. However, the effects of browsing and light on stem and crown proportions, as well as the interactive effects of browsing and light on sapling architecture, have never been examined so far. Disentangling the relative contribution of environmental determinants to sapling architecture and highlighting the patterns of co‐variations of different architectural traits can enhance the understanding of mechanisms underpinning the recruitment success of juvenile trees.

In this study, we examined the individual and interactive effects of deer browsing and light intensity on architectural traits in juvenile individuals of six tree species. To quantify browsing intensity, we developed an original index Bi, while the tested architectural traits, commonly used in studies about tree architecture, were expressed as indices derived from field measurements of sapling structural parameters. We considered not only sapling height and crown length, obvious indicators of sapling competitive strength, but also crown shape. In fact, as a result of the variety of growing conditions, the crown shape of most saplings is quite irregular, often strongly so.

Competition for light in temperate forests growing in fertile habitats is always strong, and it forces young trees to invest most of the available resources in height growth (Oliver and Larson 1990). Browsing by deer is very intense between 0.5 and 1.0 m in height (Szwagrzyk et al. 2020), and then it slows down gradually between 1.0 and 1.8 m. When ungulate herbivores are numerous, saplings that are shorter and have broader crowns have little chance to grow tall enough to eventually escape the browsing pressure.

Both BI and light intensity were calculated based on measures taken along transects set in natural and semi‐natural forests of southern Poland. The variations in light intensity were related to a disturbance gradient ranging from stand‐replacing disturbances (bark beetle outbreaks, windstorms) to small gaps created by the death of single large trees due to wind or pathogenic fungi.

Our aims are: (1) to highlight the response patterns of individual architectural parameters to variations in browsing intensity and light intensity, as well as to their combined effects; (2) to assess the relative importance of these two environmental factors in affecting these parameters. Specifically, we tested the following hypotheses:

- tree slenderness decreases with increasing browsing intensity and decreases with increasing light intensity;
- crown slenderness increases with increasing light intensity;
- crown irregularity increases with browsing intensity.

## 2 Methods

### 2.1 Study Area

Field surveys were carried out in the Tatra National Park (TNP) and the Roztocze National Park (RNP) in southern Poland. The TNP was established in 1954 over the Polish part of the Tatra Mountains and covers a surface of approximately 21,000 ha. And the elevation ranges from 900 to 2499 m a.s.l. The climate in the lower mountain zone is moderately cool, with an annual temperature of up to 5°C and an annual rainfall of 1100 mm (Hess 1996). The forests of the TNP are dominated by *Fagus sylvatica* and, in moister sites, by *Abies alba* and *Picea abies* . During the years prior to the beginning of the study, TNP had been affected by large‐scale disturbances (windthrows and bark beetle outbreaks), and the affected sites present abundant tree regeneration.

The RNP is located in the central part of the meta‐Carpathian upland known as the Roztocze Highlands, in the Southeastern part of Poland, and covers an area of approximately 8482 ha. The mean annual temperature is about 7.3°C. The yearly amplitude of the mean temperatures often exceeds 22°C. The mean annual precipitation of the Roztocze region ranges from 650 to 750 mm (Kaszewski 2008). The mixed deciduous forests of the RNP are dominated by *Fagus sylvatica* , *Abies alba*, and *Carpinus betulus* . Some almost pure silver fir forests may also be found. Unlike in TNP, in RNP, natural forest dynamics is mostly driven by regeneration processes in small or medium‐sized gaps. The soils in both study sites are dominated by relatively fertile Distric and Eutric Cambisols, with patches of poorer Leptosols and Podzols (Kaszewski 2008; Skiba 2002).

The guilds of ungulate herbivores in both national parks are similar. The most abundant species is red deer, *Cervus elaphus* ; the density of red deer in R. N. P. has been estimated at 48 individuals/1000 ha, and in the Tatra N. P. around 13 individuals/1000 ha. The second species is roe deer, *Capreolus capreolus* (13 ind./1000 ha in R.N.P. and 7 ind./1000 ha in T.N.P.). The diet of these two ungulates is mostly the same; however, because of his larger size and the higher density of his population, the red deer is the main browser in our study areas.

### 2.2 Sampling Design

Sapling architectural parameters and browsed shoots were recorded along 60 transects, 30 for each of the two study sites. Out of them, 16 transects (8 in the TNP and 8 in the RNP) were established within fenced exclosures that had been built 10–15 years earlier to protect young trees from browsing, while the remaining transects were set in areas available for ungulates to browse. This design was chosen to separately test the effect of light alone on unbrowsed saplings (within exclosures) and on browsed saplings (outside the exclosures). No signs of ungulate penetration nor browsed shoots were found within the exclosures. The transects were 30 m long and 5 m wide, with 2.5 m on each side of the transect axis, and were established in areas characterised by extensive tree regeneration. Most of them run from gap centres to the forest edges; however, some transects were established under uniformly dense forest canopy or within large openings, where the nearest forest edge was far from the transect. The rationale behind this design was to cover the entire range of light conditions in areas where forest regeneration was well developed.

A maximum number of 30 saplings (individuals from 0.5 to 3.0 m tall) of each tree species occurring in the study sites were sampled along each transect within the smallest distance from the transect axis. For less abundant species, we measured saplings over the entire transect area, and in some cases the number of saplings of an uncommon species in a transect was less than 30 individuals. We recorded the saplings of 15 tree species, but only six of them were numerous enough to be statistically analysed. They were: *Abies alba* , *Picea abies* , *Fagus sylvatica* , *Acer pseudoplatanus* , *Sorbus aucuparia* , and *Carpinus betulus* . The position of each measured sapling on the transect was recorded as distance from the beginning of the transect, measured along the transect axis. For the purpose of determining the light conditions, we also took hemispherical photographs every 5 m along the transect axis.

To determine the intensity of browsing, we measured the diameters of shoots at the point where they were browsed. We measured only shoots that were fully lignified and were at least 1 mm thick to avoid recording the signs of damage caused by invertebrates. By setting the shoot diameter threshold at 1 mm, we eliminated the tiny shoots that could have been eaten either by rodents or by birds. In each case, we started recording the browsed shoots from the treetop and measured at most 50 browsed shoots per plant. As browsing of the top shoots strongly affects the height growth of young trees (Kupferschmid, Zimmermann, and Bugmann 2013), we started the measurements of browsed twigs from the tree top and proceeded down the crown. Some tree species exhibit many lateral branches in the lower part of the crown; however, even very intense browsing of these shoots is of minor importance for saplings (Churski et al. 2022), as the lower branches are easily lost due to competition for light among young trees.

Overall, 3816 saplings of the above‐listed six species were surveyed, out of which 58% were variously browsed and 42% were unbrowsed (Table 1). Out of the unbrowsed saplings, 62% were within fences, and 38% outside them. Fieldwork was carried out in the summer of 2020 and 2021.

**TABLE 1:** Number and proportion of recorded specimens per tree species, browsed/unbrowsed type, and fenced/unfenced type.

	*Abies alba*	*Acer pseudoplatanus*	*Carpinus betulus*	*Fagus sylvatica*	*Picea abies*	*Sorbus aucuparia*	Total
Total	731	657	392	714	636	686	3816
Browsed	370 (51%)	414 (63%)	245 (62,5%)	565 (79%)	107 (17%)	532 (77%)	2233
Unbrowsed	361 (49%)	243 (37%)	147 (37,5%)	149 (21%)	529 (83%)	154 (23%)	1583
Inside the fences	241 (33%)	211 (32%)	141 (36%)	105 (15%)	143 (22%)	137 (21%)	978
Outside the fences	120 (16%)	32 (5%)	6 (1,5%)	44 (6%)	386 (61%)	17 (2%)	605

### 2.3 Variables

For each recorded sapling, we measured the following architectural parameters: (1) sapling height H; (2) the length of crown base Hb; (3) sapling basal diameter D; (4) two orthogonal crown widths. Based on these parameters we calculated in the first place: (1) crown length CL (the difference between sapling height and the height of crown base); (2) mean crown width CW (average of two crown diameters). Based on these measurements, we worked out three indices of sapling architecture: sapling slenderness (H/D), crown slenderness (CL/CW), and crown irregularity (CI). The latter is here defined as the ratio of the larger crown diameter divided by the smaller crown diameter. These indices synthesise architectural features strongly associated with tree stability, whose variations may have important implications for forest stand structure.

We also measured the diameter of browsed shoots at the point where they were damaged. Based on these measurements, a fourth index, the browsing intensity BI, was calculated as follows:

(1) $$\mathrm{BI}=\frac{\sum_{i=1}^{N}d_{i}^{2}}{D^{2}}$$

where *d* [mm] is the diameter of browsed shoots, *N* is the number of browsed shoots per plant, and *D* [mm] is the diameter of the stem at the ground level.

Saplings of the analysed tree species strongly differed in their morphological traits. In order to remove species‐specific effects, we calculated the relative values of H/D, CL/CW, and crown irregularity, scaling their values from 0 to 1 for each species separately. The further analyses were thus conducted on the whole data for all six species jointly, employing the relative values of the architectural indices.

In view of the ascertained influence of sapling height on browsing pressure (D'Aprile et al. 2020; Miller, Kinnaird, and Cummins 1982), as well as on the phenotypic expression of genetically determined morphological features (Farnsworth and Niklas 1995; Givnish 1988), we divided the analysed saplings into three height classes: short saplings (class 1) ‐up to 90 cm tall; medium saplings (class 2) between 90 and 130 cm tall; tall saplings (class 3) over 130 cm tall. The bulk of statistical analyses were conducted separately for each of these three size classes.

For each hemispherical photograph taken along the transect, we calculated the percentages of total radiation (the sum of direct radiation and diffuse radiation incident) using the Gap Light Analyser following Frazer, Canham, and Lertzman (1999). Then the relative light intensity values along each of the established transect axes were calculated by interpolation between the light values from the nearest two points where the hemispherical photographs were taken.

### 2.4 Statistical Analyses

To model the impact of BI and light intensity on the architectural indexes, we used the class of Generalised Additive Models for Location, Scale and Shape (GAMLSS) (Rigby and Stasinopoulos 2005). An advantage of the GAMLSS is that the response variable distribution can be almost any parametric distribution, and its parameters can be modelled as smooth functions of the independent variables.

We compared different distribution families based on the Akaike Information Criterion (AIC) to choose the best‐fitted model. We estimated its expected value μ modelled depending on the values of BI and light. After selecting the distribution family, we next fitted the additive components of the GAMLSS model that describe the parameters of the investigated architectonic index along with its statistical significance. When analysing the relationship between light intensity and browsing intensity (BI) and architectonic indices, a simple linear regression model was used to examine the relationship between the variables.

We also wanted to highlight which part of the variation in the architectural index values is explained by each explanatory variable, as well as by their combined effect. To this purpose we employed the variation partitioning approach (Borcard, Legendre, and Drapeau 1992; Legendre 2008), which enables splitting the variation explained independently by each variable into variation attributable purely to some response variable and shared variation assigned to both of the explanatory variables.

We also conducted separate analyses of the relationship between architectonic indices and BI for the six most numerous species. As the number of individuals in certain size classes was in some cases low, we employed robust regression as the main type of our models using the rlm function from the MASS package in R. This method was particularly suitable because the dataset comprised biological measurements that often exhibit significant variability due to inherent natural differences, measurement errors, and environmental influences. Robust regression mitigates these issues by focusing on minimising the influence of outliers and heteroscedasticity, ensuring that the estimated model. However, in the “Results” we included only the cases when the relationship calculated for a single species differed significantly from the results obtained for all species pooled together.

All calculations were performed in the R software, version 4.0.2, developed by RStudio with the gamlss package (v. 5.4.1) to perform GAMLSS model estimation (Stasinopoulos and Rigby 2007) and *vegan* (v. 2.5.7) (Oksanen et al. 2013) to perform the variation partitioning approach.

## 3 Results

**FIGURE 1:**
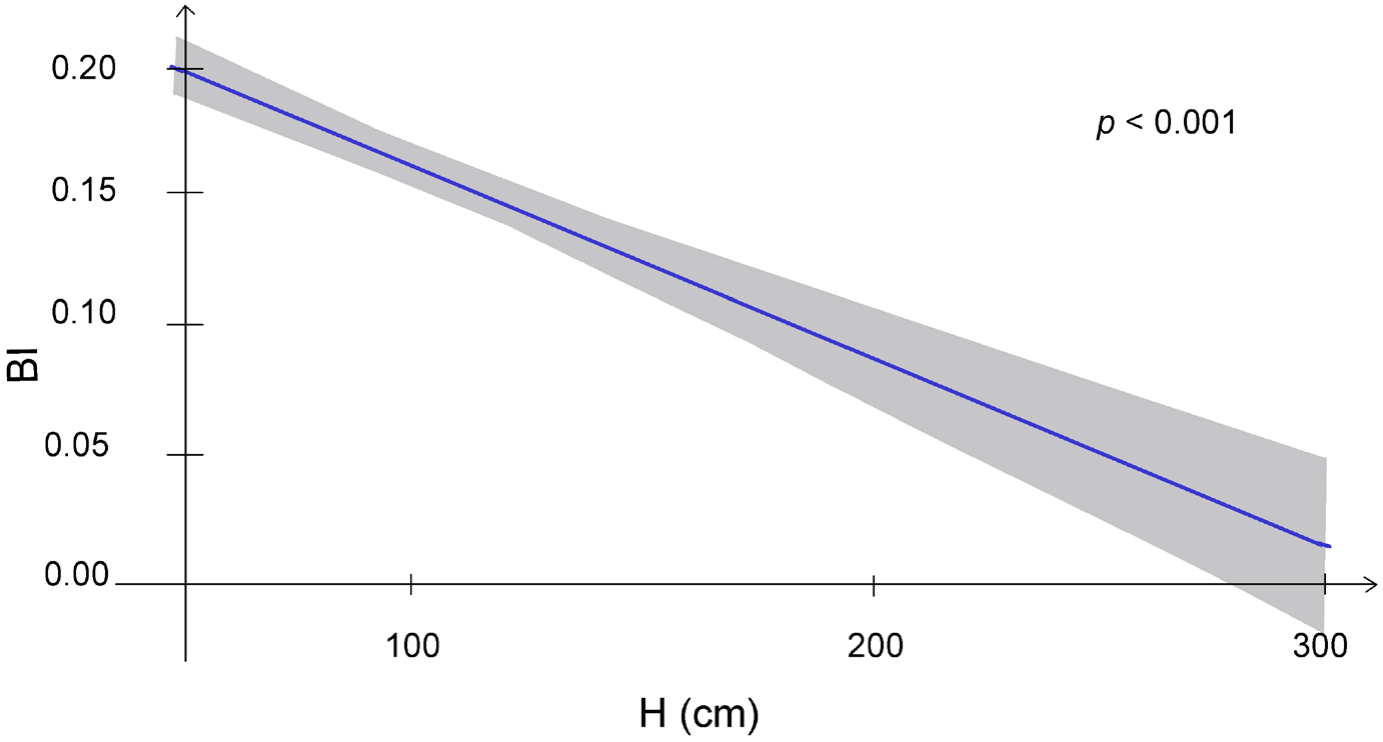
GAMLSS showing linear regression of BI on sapling height. Confidence intervals are shown in grey.

The six analysed tree species strongly differed in their architectural features, as well as in the browsing pressure exerted on them. BI exhibited a significantly negative response to sapling height, although this relationship was quite weak (R2 = 0.05) (Figure 1). Even saplings exceeding the height of 2 m are browsed, although mostly in the lower part of the crown.

**FIGURE 2:**
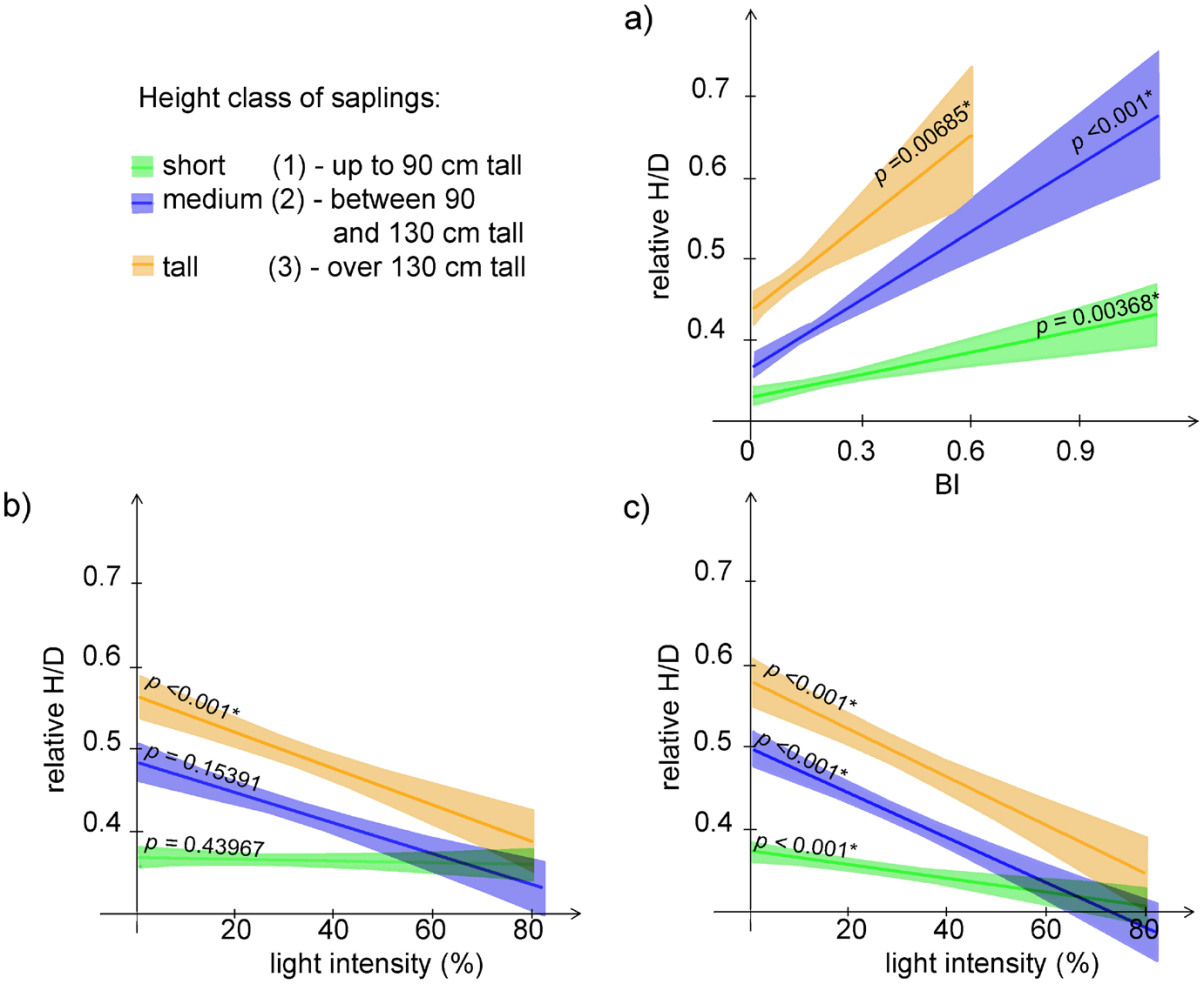
GAMLSS showing regression of H/D on: (a) BI; (b) light intensity for unbrowsed saplings; and (c) light intensity for browsed saplings. Confidence intervals are shown as coloured bands.

H/D was significantly and positively affected by BI in all sapling height classes, with the strong relationship observed for the tall and medium saplings (Figure 2 and Table 2). Light intensity exhibited a significant negative effect on H/D only for the tall unbrowsed saplings, whereas, for browsed saplings, it significantly and negatively affected H/D in all sapling height classes, with the strongest relationship found in the tall and medium saplings (Figure 2b,c and Table 2).

**TABLE 2:** GAMLSS coefficients for the regression of architectural parameters on BI and light intensity, according to the height class.

	Class1		Class2		Class3	
	Coefficient	*p*	Coefficient	*p*	Coefficient	*p*
Bi browsed saplings						
H/D	0.071	0.004	0.266	< 0.001	0.288	0.007
CL/CW	0.019	0.523	−0.035	0.482	−0.167	0.101
Crown irregularity	0.114	0.009	0.201	< 0.001	0.238	< 0.001
Light for unbrowsed saplings						
H/D	> −0.001	0.44	> −0.001	0.154	−0.01	< 0.001
CL/CW	0.001	< 0.001	0.002	< 0.001	0.002	< 0.001
Crown irregularity	0.004	< 0.001	< 0.001	0.059	> −0.001	0.042
Light for browsed saplings						
H/D	> −0.001	< 0.001	−0.003	< 0.001	−0.003	< 0.001
CL/CW	−0.001	< 0.001	−0.001	< 0.001	> −0.001	0.107
Crown irregularity	−0.002	< 0.001	> −0.001	0.024	> −0.001	0.015

*Note:* Class 1 height < 90 cm; class 2 90 ≤ height < 130 cm; class 3 height ≥ 130 cm.

**FIGURE 3:**
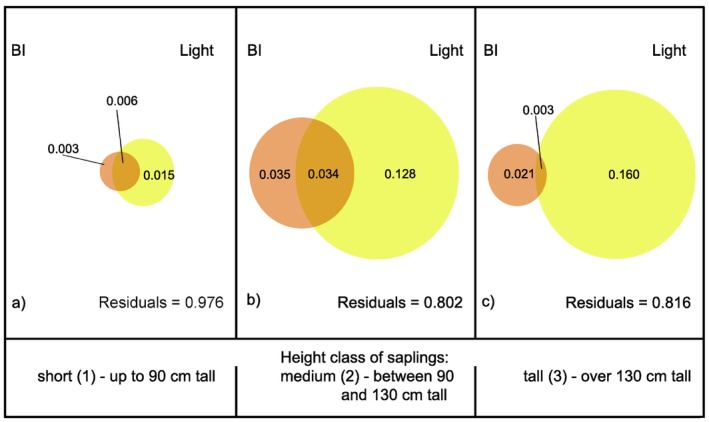
Variation partitioning for H/D explained by BI and light for: (a) short saplings; (b) intermediately tall saplings; (c) tall saplings.

Variation partitioning analysis showed that both BI and light explained a very exiguous part of variation in H/D values, especially for the short saplings, and that most of explained variation was accounted for by relative light intensity. The proportion of variation explained by the combined effect of BI and light was the highest for intermediate saplings (Figure 3b).

**FIGURE 4:**
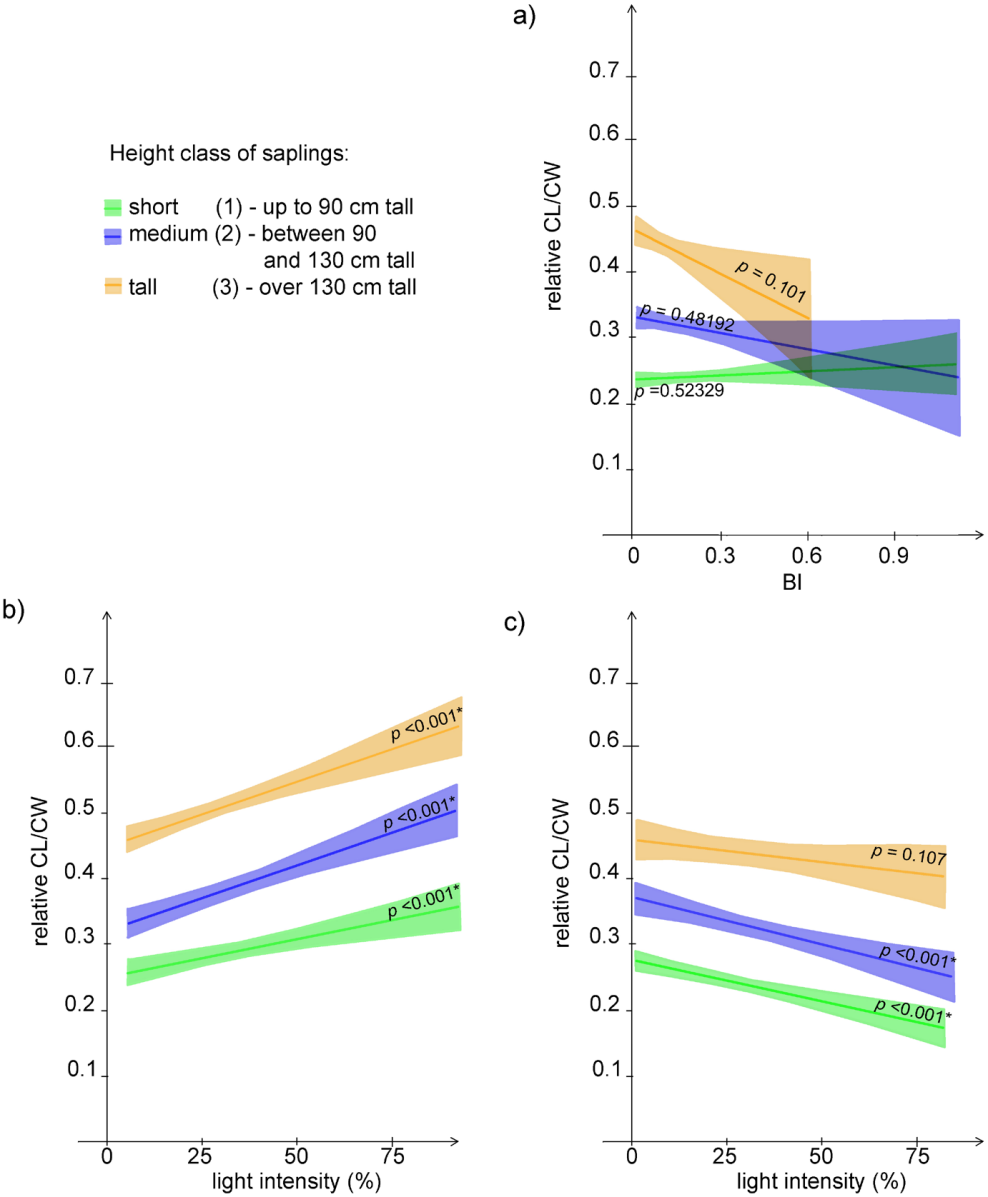
GAMLSS regression of CL/CW on: (a) BI; (b) light intensity for unbrowsed saplings; and (c) light intensity for browsed saplings.

**FIGURE 5:**
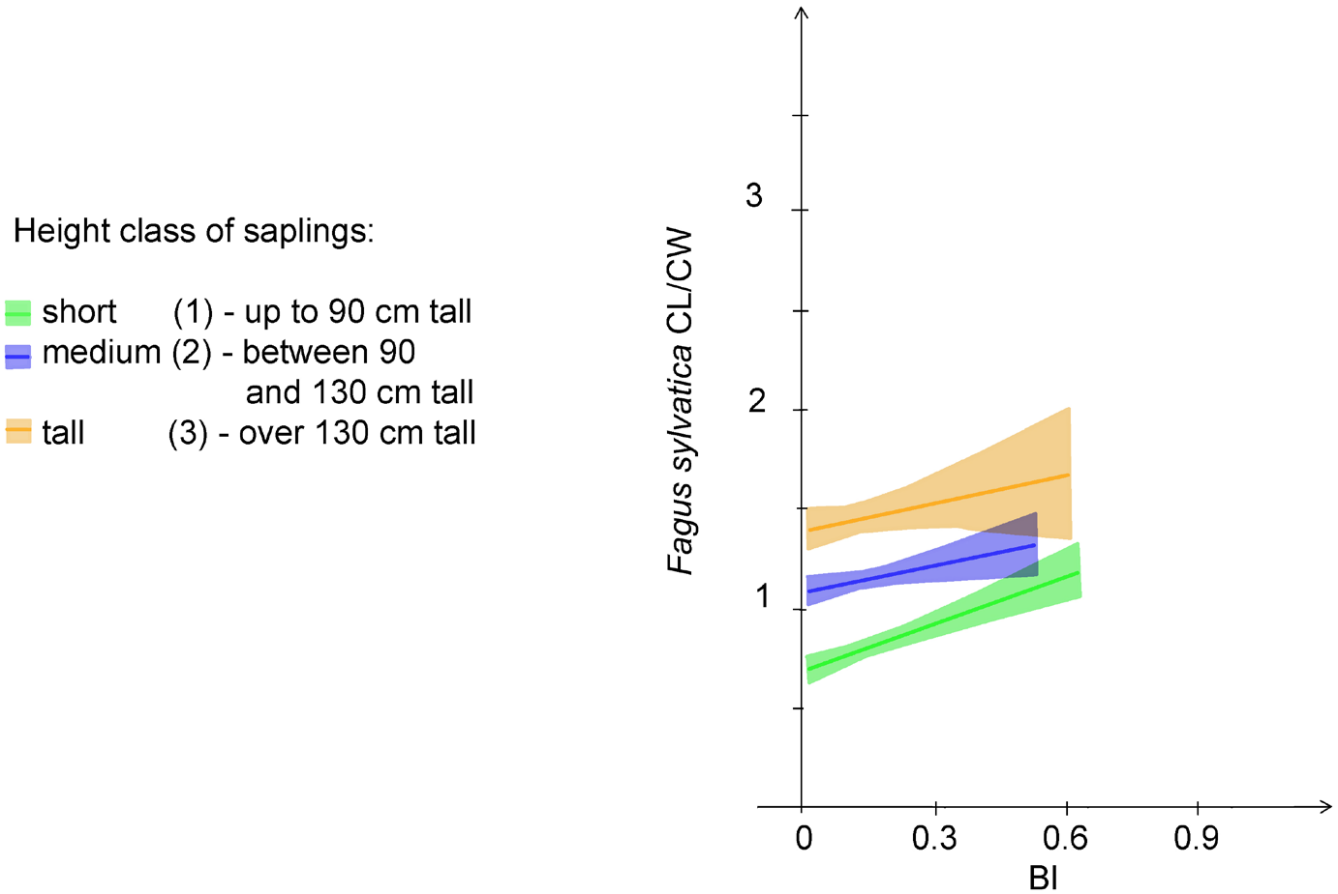
Robust regression of CL/CW on BI for browsed saplings of *Fagus sylvatica* .

CL/CW was insignificantly but negatively affected by BI for all saplings (Figure 4a and Table 2). For unbrowsed saplings, increasing light intensity significantly and positively affected CL/CW in all height classes, while for browsed saplings, increasing light intensity was associated with a significant decrease of medium and short saplings CL/CW (Figure 4b,c and Table 2). The analyses conducted separately for *Fagus sylvatica* yielded a different result (Figure 5). Crown slenderness (CL/CW) significantly increased with increasing browsing intensity (BI) for all size classes of saplings.

**FIGURE 6:**
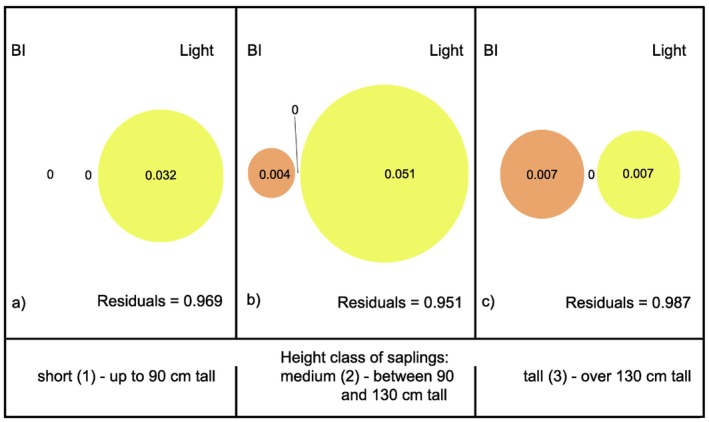
Variation partitioning for CL/CW explained by BI and light for: (a) short saplings; (b) medium saplings; (c) tall saplings.

The proportion of CL/CW variation explained by BI and light was again very exiguous, and most of it was accounted for by light, especially for short and medium saplings (Figure 6a,b). No combined effect of light and browsing was found.

**FIGURE 7:**
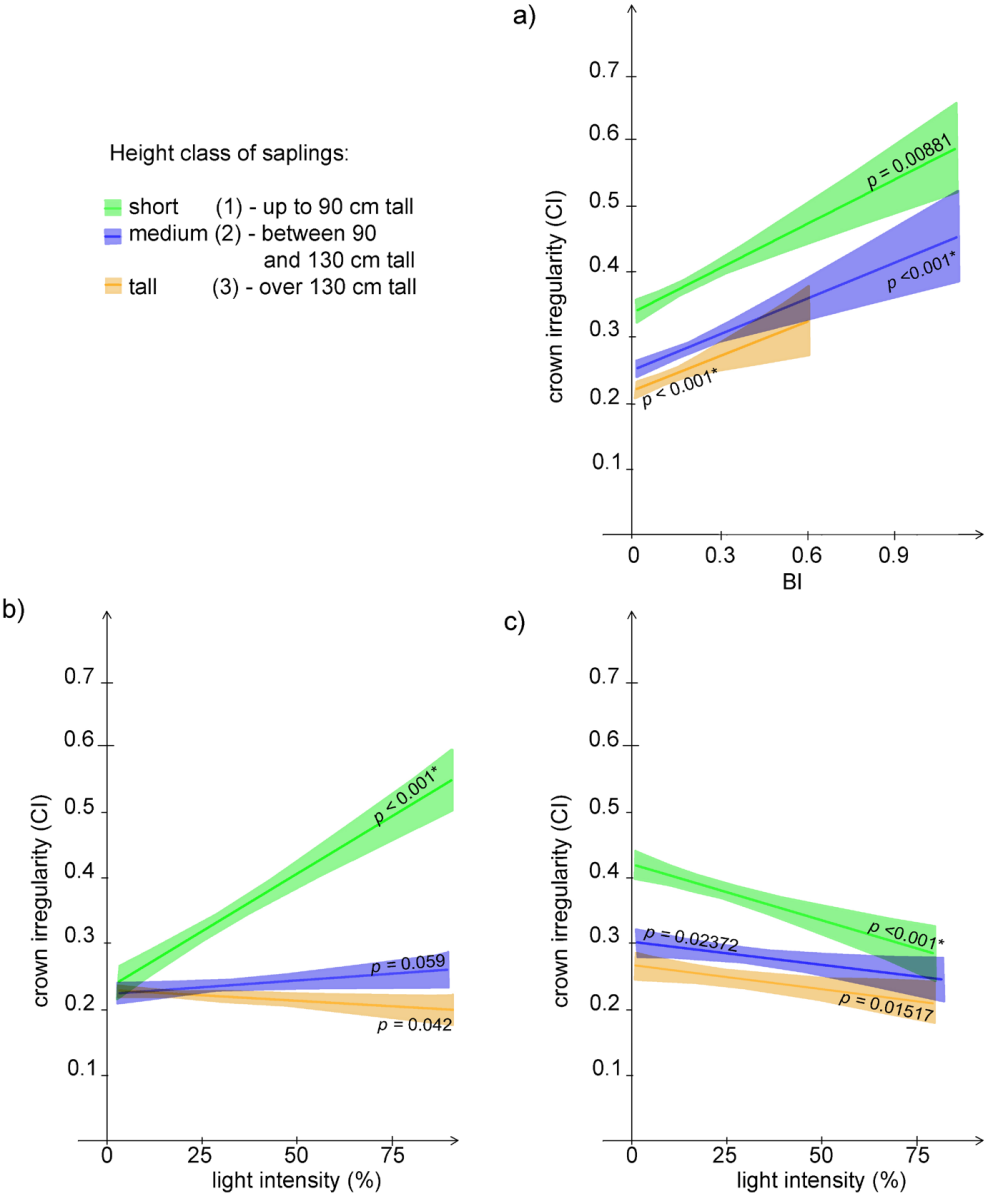
GAMLSS regression of crown irregularity (CI) on: (a) BI; (b) light intensity for unbrowsed saplings; and (c) light intensity for browsed saplings.

CI was significantly and positively affected by BI in all sapling height classes, with the strongest relationship observed for the tall and medium saplings (Figure 7 and Table 2). For unbrowsed saplings, the relationship between light and crown irregularity was inconsistent among the size classes, with a significant positive relationship only in short saplings. In browsed saplings, CI was significantly and negatively affected by light intensity in all sapling height classes, with the strongest relationship being observed for the short saplings (Figure 7b,c and Table 2).

**FIGURE 8:**
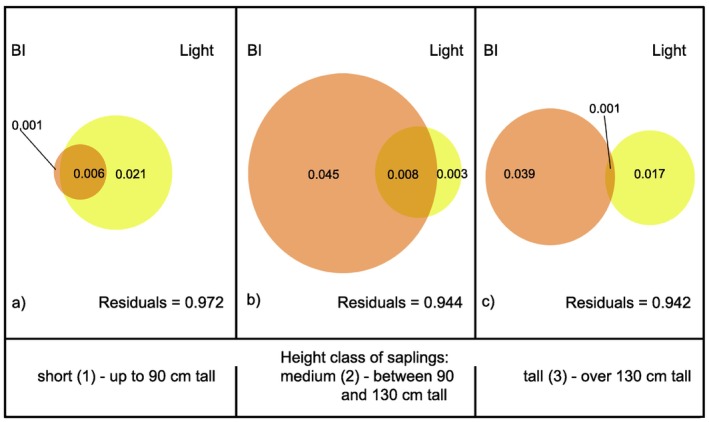
Variation partitioning for crown Irregularity Index explained by BI and light for: (a) short; (b) medium; (c) tall saplings.

As in the case of H/D and CL/CW, the proportion of explained variation in crown irregularity values was quite small, but, unlike the other two indices, for medium and tall saplings it was mainly accounted for by Bi, while for the short saplings the main driver remained light (Figure 8).

## 4 Discussion

Browsing by ungulates is generally assumed to concentrate on the upper parts of the sapling crowns, which causes sapling vertical growth to slow down or, at high intensity, to stop altogether. Meanwhile, the issue of whether or not this translates into a shift in resource allocation toward lateral expansion, with stem enlargement and development of lateral shoots, is less clear. The net effect on sapling development, architecture, and, ultimately, survival depends on the species‐specific branching pattern and ecological strategy, that is, on the ability to compensate for the biomass removal and to tolerate prolonged shading, but also on exogenous factors, like light availability (Krueger et al. 2009). The chances of effectively compensating for browsing‐induced damage are higher for those species producing many lateral shoots (Churski et al. 2022), as well as for individuals growing under favourable light conditions (under large canopy gaps or in larger open areas) (Kupferschmid, Zimmermann, and Bugmann 2013; Kupferschmid, Wasem, and Bugmann 2014a). Besides, not all saplings are browsed in the upper part of their crowns. Our results showed that the mechanisms underpinning the relationship between exogenous factors and allometric proportions are fundamentally height‐dependent. Instead of decreasing with increasing browsing pressure, as we expected, H/D exhibited an increasing trend, which was the strongest in the case of tall saplings. This can be accounted for by the variation in browsing modes determined by sapling heights.

In short, saplings browsing indeed concentrates in the upper part of the crown, thus substantially reducing their capacity for increasing in height. However, due to reduced leaf area, the diameter growth in heavily browsed saplings is also reduced, so as a net result, crown slenderness exhibits a slight increase along with increasing BI. In tall saplings, browsing affects the side branches rather than the top shoots and thus reduces the crown size and leaf area, without affecting the height growth, because in young trees the height growth has a higher priority in assimilate partitioning compared to the diameter growth (Dizès et al. 1997). Therefore, the tall saplings under browsing pressure grow slenderer than the less browsed individuals.

The browsing‐induced increase in sapling slenderness may be interpreted as an attempt of saplings to escape further browsing and would thus express a strong adaptive reaction of juvenile trees to browsing. Similar findings were reported by Häsler, Senn, and Edwards (2007), Kupferschmid and Bugmann (2013); Kupferschmid, Zimmermann, and Bugmann (2013); and Kupferschmid, Wasem, and Bugmann (2014a), who observed that the height growth of *A*. *alba* was not reduced in saplings growing in relatively good light conditions, as the browsed terminal shoots were replaced by the flagging up of lateral shoots. On the contrary, the height increment of saplings growing in the shade could not be compensated for because of the limited photosynthesis. On the other side, Ammer (1996) and Kupferschmid, Wasem, and Bugmann (2014b) reported that browsed saplings were consistently shorter than unbrowsed ones. The increased slenderness of intensively browsed individuals could be interpreted as the last effort of saplings to escape browsing. If this effort is not successful, the sapling may not survive.

The effect of light intensity on sapling allometric proportions turned out to be even more markedly height‐dependent than that of browsing. Light intensity affected sapling slenderness only in tall saplings, which tended to become stouter along the light gradient. The effect of light on sapling slenderness being limited to the higher individuals may be accounted for by assuming that short and medium saplings are affected by other factors, for example, competition by neighbours, counteracting the tendency of saplings to reduce the height/diameter ratio under favourable light conditions. Ametzegui and Coll (2011) reported a clear decrease of sapling slenderness along with increasing light intensity, and Orman et al. (2021) observed no response of sapling slenderness to increasing irradiance. In fact, both these studies examined light‐driven changes in sapling architecture without considering sapling height. Testing for the impact of exogenous factors on sapling architecture depending on height classes allows us to highlight the variations of such response due to height‐related changes in micro‐environmental conditions. On the other hand, it makes it difficult to pinpoint a general pattern and to provide a single, coherent answer to the question of how architectural parameters react to changes in environmental drivers.

The superimposition of the effect of browsing on the effect of light radically changed the pattern. The effect of increasing light intensity on browsed individuals caused a decrease of sapling slenderness in all sapling height classes, although for short saplings such a trend was quite weak. This means browsing, by “pruning” saplings, loosened competition, which caused slenderness to decrease along with increasing light intensity.

Our results suggest that the impact of browsing on architectural parameters does not depend solely on its intensity and extent but also on its mode. Crown slenderness (CL/CW) exhibited insignificant response to BI in all saplings. This may at first look surprising, since browsing pressure is higher in short and medium saplings, but it might be explained by taking into account the implication of sapling height on browsing mode. The crowns of the lower saplings are browsed, especially in their upper half. This keeps the CL/CW ratio quite constant along with increasing browsing intensity. Instead, the crowns of the tall saplings are browsed mostly from below. The concentration of the browsing pressure in the lower part of the crown may reduce the crown length and thus decrease the CL/CW ratio.

However, the analysis conducted separately for *Fagus sylvatica* showed a consistent pattern of increase of crown slenderness along with browsing intensity in all size classes of saplings. *Fagus* is among the least browsed species in our data set (Szwagrzyk et al. 2020). It is also, along with *Carpinus betulus* (Churski et al. 2022), characterised by sympodial growth and a high number of lateral branches. The results suggest that moderate browsing pressure constricts the lateral expansion of tree crowns without slowing down the height growth. That could contribute to the successful recruitment of beech saplings to higher size classes and dominance of this species in the young generation of forest trees (Nagel et al. 2015).

The response of crown slenderness to light intensity was in line with our hypothesis. We assumed that, since the crowns of saplings growing in very poor light conditions are relatively flat, or even umbrella‐shaped (Oliver and Larson 1990), crown slenderness should increase along with increasing light intensity, which was confirmed by our results, showing a consistent increase of crown slenderness along with light in all sapling height classes.

As in the case of the relationship between H/D and BI, browsing by ungulates reversed the response of CL/CW to the light intensity gradient. For browsed saplings, increasing light intensities induce a decrease of CL/CW for short and medium saplings. Here again, the browsing may have loosened competition by pruning neighbouring saplings, which made light fully available and caused a decrease of CL/CW with increasing light. In the case of the tall saplings, this relationship is barely outlined, because taller saplings are less intensively browsed, and, consequently, the “pruning effect” is reduced; competition for lateral crown expansion remains strong, and since light is hardly accessible, crowns cannot develop laterally.

The response of crown irregularity to BI supported our hypothesis, being significant and positive for all the medium and tall height classes. However, this positive response turned out to be stronger for the tall and medium saplings, which was surprising, since browsing pressure was stronger on short saplings. Meanwhile, crown irregularity increased with increasing light intensity, which was in contrast with our hypothesis. This may be accounted for by calling upon competitive interactions among neighbouring saplings. The higher the light intensity, the higher the crowding effects, which intensifies the competition for space and determines the observed increase in crown irregularity. When the response of crown irregularity to light was analysed in browsed saplings, the trend was the opposite. Probably the “pruning” effect of browsing on neighbouring plants outweighs the effects of competition for light and leads to a decrease of irregularity. We assume that, in the absence of competition from neighbouring saplings and of browsing pressure, crown irregularity should decrease along with increasing light intensity, as reported by Brisson (2001).

In spite of the marked trends highlighted by the GAMLSS, the share of variation explained by the variation partitioning analyses turned out to be quite small for all tested architectural indices, especially for irregularity and crown slenderness. This means environmental factors other than browsing and light intensity play a greater role in shaping sapling architecture, the most likely candidates being competition from neighbours, microclimate, and local soil conditions. The considerable role of competition by neighbours was already highlighted by several studies (Brisson 2001; Fichtner et al. 2013; Lintuinen and Kaitaniemi 2010; MacFarlane and Kane 2017; Thorpe et al. 2010), however, none of them specifically tested the share of variation in architectural parameters explained by competitive interactions. The assessment of the relative contribution of individual environmental drivers to variations in sapling architectural traits, by directly answering the question of the extent of the environmental control on tree architectonic variability, represents a fundamental tool to understand the complex interaction between genetically predetermined organisms and the selective pressure of their habitat and should thus be systematically carried out.

As for the simultaneous effect of browsing and light intensity, GAMLSS models and variation partitioning yielded inconsistent results. According to the latter, the interaction between light and browsing intensity played no role in explaining variations in CL/CW, while the GAMLSS models showed that the responses of CL/CW to the light gradient were opposite for browsed and unbrowsed saplings, and both trends were statistically significant. That implies that browsing and light combined exert a different effect on crown slenderness than light alone. In the case of crown irregularity, according to the Variation Partitioning analyses, the simultaneous effect of browsing and light intensity played a slightly minor role for short saplings than for intermediate ones. Meanwhile, GAMLSS models showed that it was just short saplings that underwent the stronger change when comparing browsed and unbrowsed saplings, the response of crown irregularity to light diametrically reversing when browsed saplings were tested.

In general, most of the relationships highlighted in this study were relatively weak, although significant. That is because we analysed together data about six tree species, the ones that were numerous enough to allow for meaningful analyses. However, each of those species represented a different growth strategy and reacted to the damage caused by browsing in a different way. These reactions are sometimes opposite, so the net effects obtained for pooled data are much weaker than for individual species. Previous studies about the effects of light and browsing upon juvenile tree architecture have usually focused upon one or two tree species (Kupferschmid, Zimmermann, and Bugmann 2013; López‐Sánchez et al. 2021), which makes it likely to obtain consistent patterns. When analysing more species together, one should be aware of the difficulty of highlighting significant patterns. Our study suggests that a large share of browsing and light effects upon tree morphology is inherently species‐specific and that highlighting strong patterns when studying phenomena based on a large species pool might be difficult.

## Author Contributions

**Cecilia Cacciatori:** investigation (equal), writing – original draft (lead), writing – review and editing (lead). **Anna Gazda:** conceptualization (equal), data curation (lead), formal analysis (equal), investigation (equal), methodology (equal), validation (equal), visualization (supporting), writing – original draft (equal), writing – review and editing (equal). **Jan Bodziarczyk:** conceptualization (equal), investigation (equal), writing – review and editing (equal). **Kacper Foremnik:** investigation (equal), writing – review and editing (equal). **Aklilu B. Madalcho:** investigation (equal), writing – review and editing (equal). **Zbigniew Maciejewski:** investigation (equal), writing – review and editing (equal). **Remigiusz Pielech:** investigation (equal), writing – review and editing (equal). **Andrzej Tomski:** conceptualization (equal), formal analysis (equal), methodology (equal), software (lead), visualization (lead), writing – original draft (equal), writing – review and editing (equal). **Antoni Zięba:** investigation (equal), writing – review and editing (equal). **Tomasz Zwijacz‐Kozica:** investigation (equal), writing – review and editing (equal). **Jerzy Szwagrzyk:** conceptualization (lead), data curation (equal), formal analysis (equal), funding acquisition (lead), investigation (lead), methodology (lead), project administration (lead), resources (equal), supervision (lead), validation (equal), writing – original draft (equal), writing – review and editing (lead).

## Conflicts of Interest

The authors declare no conflicts of interest.

## Supporting information


Data S1.

## Data Availability

Data can be accessed from the figshare Digital Repository https://figshare.com/s/c1e24d21e6a2ed0b6b57 (Gazda, Szwagrzyk 2024).
