# Supplementary material for: Deer Browsing Increases Stem Slenderness and Crown Irregularity and Modifies the Effects of Light Gradients on Architecture of Forest Tree Saplings
Source: Ecol Evol. 2025 Jan 20;15(1):e70837. doi: 10.1002/ece3.70837 (PMC11745818; doi:10.1002/ece3.70837)

Supplementary material

**Supplemental Figure 1.** Distribution of architectural indices based on raw values for the six tree species: a) H/D (sapling slenderness); b) CL/CW (crown slenderness); c) Irregularity (crown irregularity)


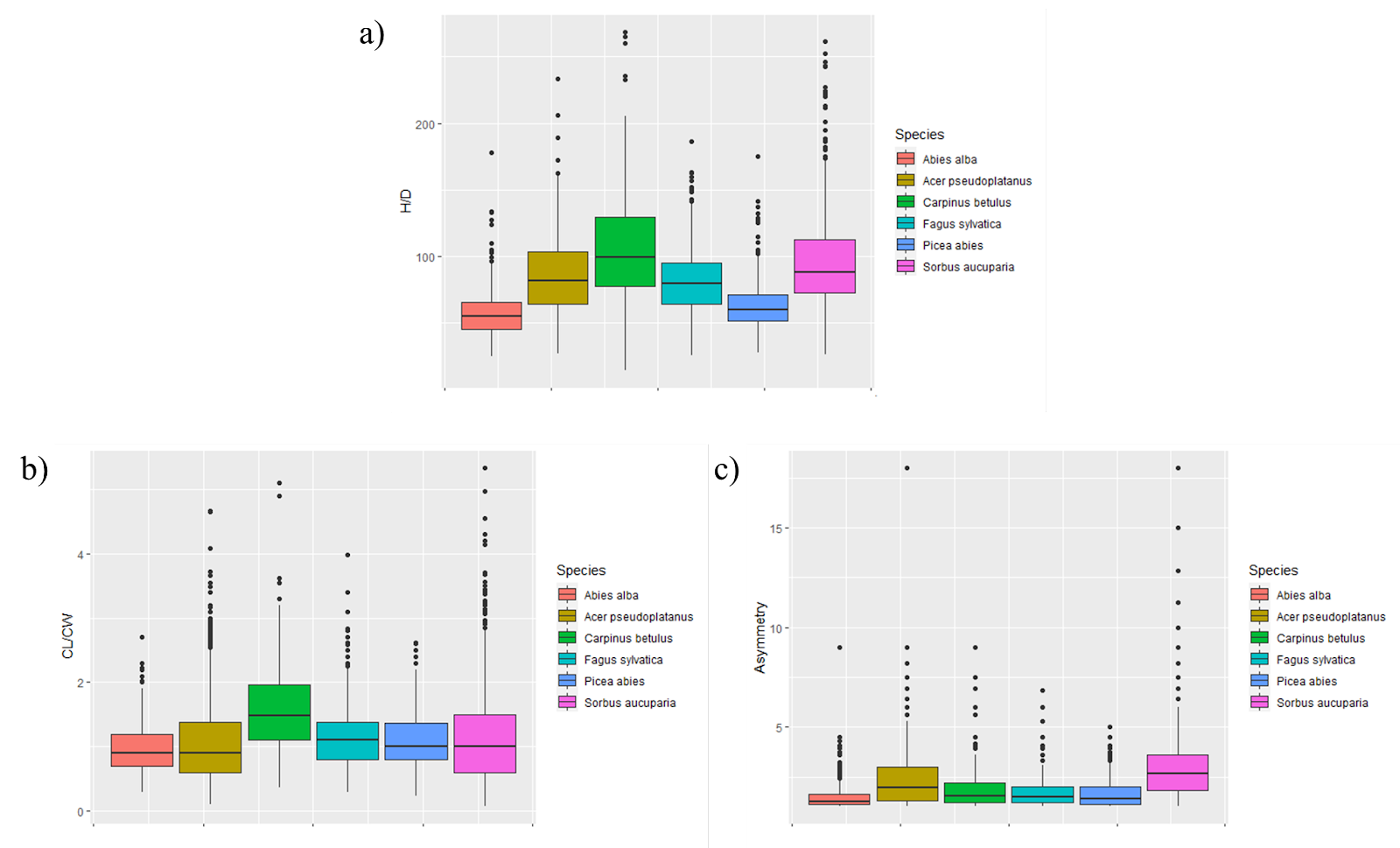


Irregularity

**Supplemental Figure 2.** Distribution according to tree species of a) Bi and b) light intensity.


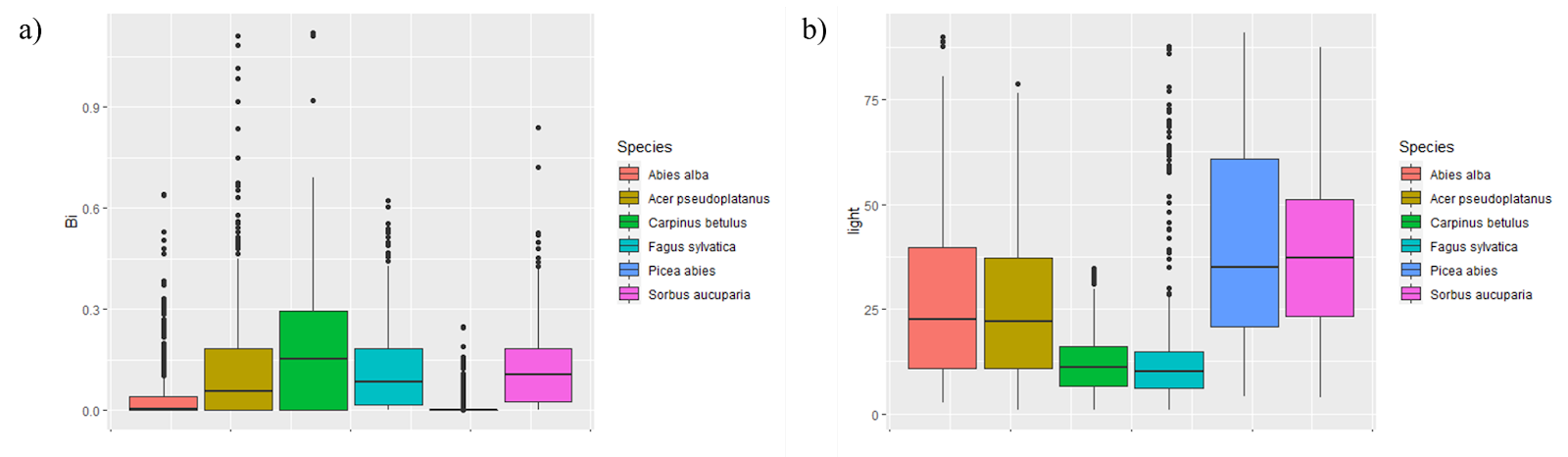

Supplement: Supplementary file 1 — Data S1. [file ECE3-15-e70837-s001.docx]
